# Crosslinked Hyaluronic Acid with Liposomes and Crocin Confers Cytoprotection in an Experimental Model of Dry Eye

**DOI:** 10.3390/molecules26040849

**Published:** 2021-02-06

**Authors:** Sawan Ali, Sergio Davinelli, Rita Mencucci, Franco Fusi, Gianluca Scuderi, Ciro Costagliola, Giovanni Scapagnini

**Affiliations:** 1 Department of Medicine and Health Sciences “V. Tiberio”, University of Molise, 86100 Campobasso, Italy; sergio.davinelli@unimol.it (S.D.); ciro.costagliola@unimol.it (C.C.); giovanni.scapagnini@unimol.it (G.S.); 2 Eye Clinic, Department of Neurosciences, Psychology, Pharmacology and Child Health, University of Florence, 50134 Florence, Italy; rita.mencucci@unifi.it; 3 Department of Experimental and Clinical Biomedical Sciences “Mario Serio”, University of Florence, 50134 Florence, Italy; franco.fusi@unifi.it; 4 Ophthalmology Unit, NESMOS Department, St. Andrea Hospital, Sapienza University of Rome, 00189 Rome, Italy; gianluca.scuderi@uniroma1.it

**Keywords:** dry eye, hyaluronic acid, crocin, liposomes, anti-inflammatory, antioxidants

## Abstract

Dry eye disease (DED) is a multifactorial condition caused by tear deficiency and accompanied by ocular surface damage. Recent data support a key role of oxidative and inflammatory processes in the pathogenesis of DED. Hyaluronic acid (HA) is widely used in artificial tears to treat DED by improving ocular hydration and reducing surface friction. Crocin (Cr), the main constituent of saffron, is a renowned compound that exhibits potent antioxidant and anti-inflammatory effects. The present study was undertaken to assess the viscosity and muco-adhesiveness of a photoactivated formulation with crosslinked HA (cHA), Cr, and liposomes (cHA-Cr-L). Our aim was also to evaluate whether cHA-Cr-L may exert cytoprotective effects against oxidative and inflammatory processes in human corneal epithelial cells (HCECs). Viscosity was measured using a rotational rheometer, and then the muco-adhesiveness was evaluated. Under hyperosmolarity (450 mOsm), the HCECs were treated with cHA-Cr-L. Interleukin-1β (IL-1β) and tumor necrosis factor α (TNFα) were quantified by quantitative real-time polymerase chain reaction (RT-qPCR). The levels of reactive oxygen species (ROS) were measured using the DCF assay. The combined action of cHA-Cr-L produced a higher viscosity and muco-adhesiveness compared to the control. The anti-inflammatory effect of cHA-Cr-L was achieved through a significant reduction of IL-1β and TNFα (*p* < 0.001). The results also showed that cHA-Cr-L reduces ROS production under conditions of hyperosmolarity (*p* < 0.001). We conclude that cHA-Cr-L has potential as a therapeutic agent in DED, which should be further investigated.

## 1. Introduction

Dry eye disease (DED) is defined as a multifactorial disease caused by tear deficiency, and it is often accompanied by ocular surface damage [1]. Recent evidence supports the key role of inflammatory and oxidative processes in the pathogenesis of DED. In fact, high levels of reactive oxygen species (ROS) and increased inflammatory markers are reported in the tear film of DED patients [2,3]. In DED, inadequate tear production or excessive tear evaporation leads to tear film instability and hyperosmolarity with subsequent ocular surface inflammation [4]. Eye inflammation, in turn, can affect the dysfunction of the ocular surface and its associated structures, leading to further tear deficiency and damage [5]. Additionally, oxidative stress causes macromolecular alterations and damage to ocular epithelial cells and lacrimal glands, exacerbating inflammation [2,6].

Artificial tears play a crucial clinical role in treating DED and are associated with several beneficial effects on the tear film’s physical characteristics and ocular surface epithelium [7]. Although new treatments are becoming available, artificial tears are still the first-line treatment of DED. One of the main challenges associated with conventional eye drops is the short retention time on the ocular surface. After instillation, there is drainage of the components, mainly due to blinking and lachrymation, which lower the effectiveness [8,9].

Hyaluronic acid (HA) is a glycosaminoglycan widely distributed throughout connective, epithelial, and neural tissues, with an excellent water-holding capacity. HA is commonly used in artificial tears to treat DED symptoms as it enhances ocular surface hydration and minimizes friction, dependent on its molecular weight and viscosity [10]. Furthermore, HA counteracts ROS production, acts as a cytoprotective agent, and exerts long-term beneficial effects on corneal epithelium regeneration [11,12]. However, eye drops with HA lose viscosity as a function of time, reaching DED patients with decreased activity and effectiveness [13]. Covalent cross-linked HA (cHA) is a more viscoelastic material, enhancing the contact time with the ocular surface and has an improved physical and chemical stability compared to linear HA [14]. Liposomes are well-tolerated lipidic elements that reduce aqueous evaporation by increasing tear film stability. The clinical efficacy of liposomes is well established, mainly due to phosphatidylcholine and cholesterol, which play an essential role in developing a monolayer surface and reducing surface tension [15,16].

Crocin (Cr), the main constituent of saffron, is a water-soluble carotenoid that can interact with light in the wavelength range of 380–510 nm (violet-blue and green spectral colors). Cr undergoes a reversible photochemical reaction that generates a balance between cis-trans forms, with a concentration of about 20% of 13-cis-Cr at the photo-equilibrium. This photoactivation allows Cr, in an aqueous solution, to establish non-covalent interactions with different molecular structures, increasing the degree of cross-linkage and, consequently, viscosity and mucoadhesive properties [17]. Moreover, Cr exerts several cytoprotective properties, including anti-inflammatory and antioxidant activities [18,19].

Therefore, the purpose of this study was to assess the viscosity and mucosal adhesiveness of cHA with Cr and liposomes (cHA-Cr-L). Our aim was also to evaluate whether cHA-Cr-L may exert cytoprotective effects against oxidative and inflammatory processes in human corneal epithelial cells (HCECs).

## 2. Results

**Figure 1:**
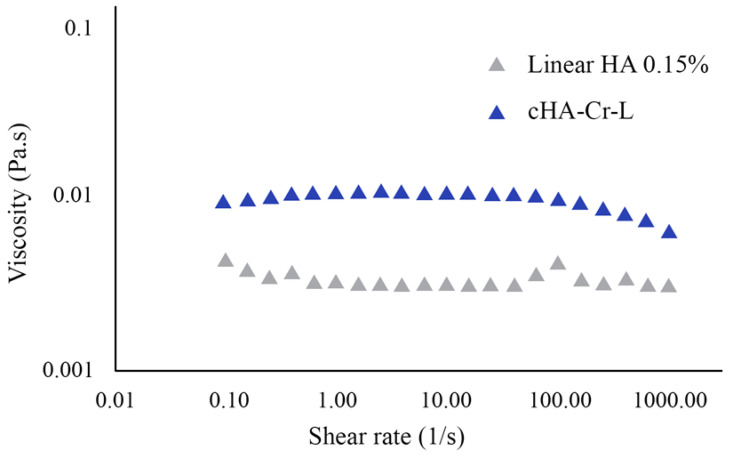
Shear viscosity of cHA-Cr-L compared to linear hyaluronic acid (HA) 0.15%.

**Figure 2:**
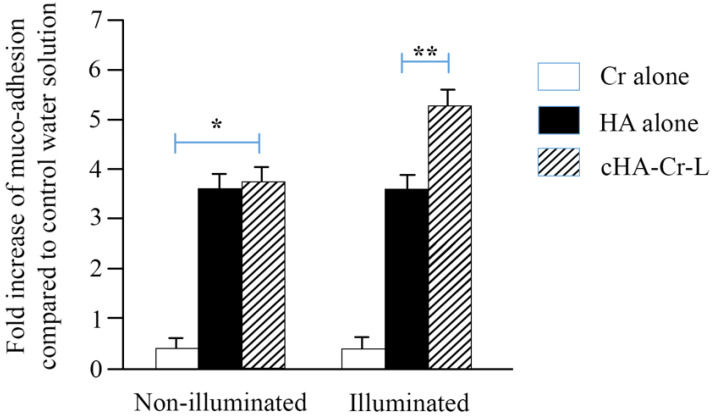
Muco-adhesivity of cHA-Cr-L, Cr, and HA. Results are expressed as fold increase of muco-adhesive index compared to control. * *p* < 0.01 cHA-Cr-L vs. Cr alone; ** *p* < 0.01 cHA-Cr-L vs. HA alone.

We used a rotational rheometer to evaluate the viscosity of cHA-Cr-L and its interaction with mucin. The results depicted in Figure 1 show that photoactivated cHA-Cr-L has a higher shear viscosity compared to linear HA 0.15%, with values of 0.0112 ± 0.0006 and 0.0033 ± 0.0004 Pa.s at 1 s^−1^, and 0.0099 ± 0.0004 and 0.0042 ± 0.0002 Pa.s at 100 s^−1^ shear rates, respectively (Table 1). At high shear rates, cHA-Cr-L slightly shifted, exhibiting shear thinning behavior. Furthermore, cHA-Cr-L exhibited a high muco-adhesiveness under illuminated conditions, as its interaction with mucin was seven-fold higher than Cr alone (*p* < 0.01) (Figure 2).

**Figure 3:**
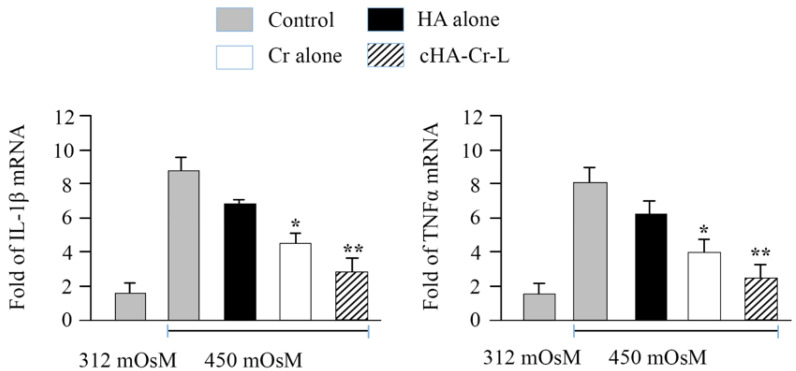
mRNA levels of IL-1β and TNFα in HCECs exposed to hyperosmotic media and treated with cHA-Cr-L, Cr, and HA. Cr and cHA-Cr-L vs. control * *p* < 0.01; ** *p* < 0.001.

Then, we examined the anti-inflammatory effect of cHA-Cr-L under hyperosmolarity in HCECs. Figure 3 shows the mRNA levels of IL-1β and TNFα in HCECs incubated with cHA-Cr-L, HA, and Cr. Treatment with 450 mOsM medium increased mRNA expression of IL-1β and TNFα to 8.68 ± 0.79 and 8.05 ± 0.94 fold, respectively, compared with normal control cells (312 mOsM). However, the expression of these proinflammatory cytokines significantly decreased to 4.58 ± 0.6 and 3.98 ± 0.74 fold (*p* < 0.01), respectively, in HCECs at 450 mOsM treated with Cr. The cytokines were further downregulated to 2.84 ± 0.79 and 2.49 ± 0.76 fold (*p* < 0.001), respectively, when treated with cHA-Cr-L. Conversely, a non-significant decline was seen in HA-treated cells. These results suggest that cHA-Cr-L has a potent suppressive effect on inflammatory mediators at mRNA levels.

**Figure 4:**
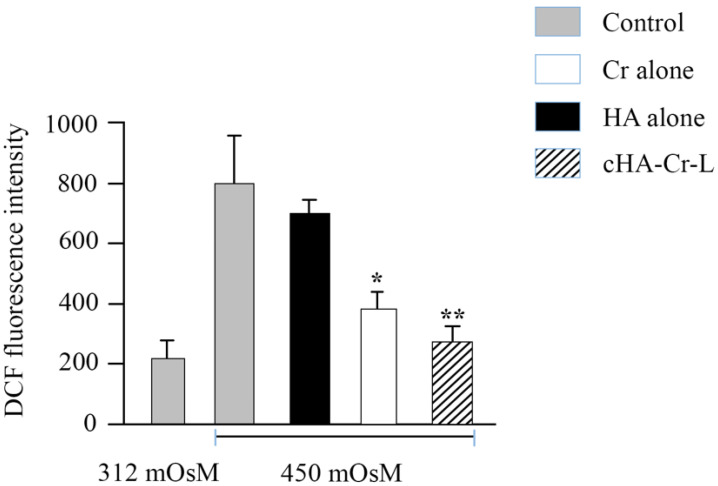
Levels of ROS in HCECs exposed to hyperosmotic media and treated with cHA-Cr-L, Cr, and HA. Cr and cHA-Cr-L vs. control * *p* < 0.01; ** *p* < 0.001.

Finally, oxidative stress was determined by quantifying ROS levels in HCECs. This was accomplished by DCF assay, which is used to detect all ROS forms generated during cell metabolism. DCF fluorescence intensity measurement revealed that hyperosmotic stress markedly stimulated intracellular ROS production. However, Cr and cHA-Cr-L treatment significantly reduced ROS levels from 798 ± 158 to 383 ± 55 and 275 ± 52, respectively (*p* < 0.01), while HA treatment resulted in a non-significant decline in DCF intensity (Figure 4).

## 3. Discussion

In this study, we tested the rheological properties and cytoprotective effects of cHA-Cr-L on HCECs exposed to hyperosmolarity. The data demonstrated that cHA-Cr-L has an enhanced viscosity with shear thinning behavior and a strong muco-adhesive property. The results also showed that cHA-Cr-L has direct antioxidant and anti-inflammatory effects in an experimental model of DED. High viscosity at low shear rates improves hydration of the ocular surface between blinks, and decreased viscosity at high shear rates is beneficial for ocular comfort during blinking and potentially reduces friction-related inflammation. Artificial tears that display low viscosity at low shear rates are more likely to drain or evaporate and are usually used in thick mucus secretion cases. In contrast, excessive viscosity due to shear thickening or resistance to shear-thinning may result in blurred vision [20]. Furthermore, the muco-adhesive capacity of a formulation prolongs the adhesion time with the ocular surface, allowing a sustained delivery and reducing the frequency of administration [21]. Eye drops with low adhesion may not retain the required time to relieve DED symptoms [10]. The interaction between adhesive compounds and mucin is generally formed by physical entanglements, van der Walls bonds, electrostatic forces, and hydrogen bonds [22]. The enhanced viscosity and muco-adhesive property of cHA-Cr-L would help to develop a protective layer on the ocular surface, improving lubrication and hydration, and reducing friction [23]. Thus, the results of our study provide a rationale for the use of cHA-Cr-L as a potential agent to improve the rheological characteristics of eye drops.

Hyperosmolarity of the muco-aqueous tear layer is one of the major etiological factors in ocular surface inflammation [21,24]. In DED, tear hyperosmolarity is associated with an increased expression of proinflammatory cytokines, chemokines, and adhesion molecules, resulting in ocular surface damage [25]. We confirmed the inflammatory role of hyperosmolarity in our DED experimental model, observing an increase of proinflammatory cytokines in HCECs exposed to hyperosmolarity. Interestingly, we also observed a significant decline of IL-1β and TNFα after the treatment with cHA-Cr-L. These results confirmed previous findings obtained in similar experimental conditions but using a single compound, such as HA or Cr [26,27,28]. In our study, the combined action of cHA-Cr-L appears more effective than the response achieved by the single compounds, indicating a synergistic effect between cHA and Cr that may be useful to suppress inflammatory processes associated with DED.

The human eye is particularly vulnerable to oxidative stress, mainly due to constant exposure to sunlight, high metabolic activities, and oxygen tension [29]. Several antioxidants are present in the tear, protecting the ocular surface; however, instability in the tear film leads to ROS overproduction [6,29]. An association between ROS overproduction, lipid and protein oxidation, and inflammatory processes have been reported in DED patients and animal models [30,31,32]. Scavenging ROS would prevent oxidative damage and, therefore, potentially reduce DED symptoms. ROS overproduction in hyperosmolarity-stimulated HCECs was effectively diminished by cHA-Cr-L, as determined by DCF fluorescent intensity. This is consistent with the antioxidant activities of Cr, which is known to neutralize free radicals and convert them into stable forms through its hydroxyl and sugar moieties [19,33]. Cr is also reported to inhibit lipid peroxidation, increase superoxide dismutase (SOD) levels, and reverse the harmful effects of oxidative stress [33,34]. These data and our results reveal that Cr potentially protects the human corneal epithelium from hyperosmolarity-induced oxidative damage.

## 4. Materials and Methods

### 4.1. Viscosity Measurements

Viscosity was measured using a discovery hybrid rotational rheometer (DHR-2) (TA Instruments, Milan, Italy) equipped with a 60 mm diameter 1° cone-plate geometry at 20 °C. cHA-Cr-L (Lumixa^®^; FB-Vision, Ascoli Piceno, Italy) is a formulation based on a 6% buffered L dispersion (water, lecithin, and propylene glycol), of which 10% is L with 0.15% cHA and Cr. The shear viscosity of cHA-Cr-L and linear HA 0.15% was obtained for shear rates ranging from 0.1 to 1000.0 s^−1^. A flow curve, which is the dynamic viscosity as a function of the shear rate, was obtained as the mean of three measurements. Before the measurement, cHA-Cr-L was left in direct daylight for 30 min.

### 4.2. Muco-Adhesion Measurements

The muco-adhesiveness of the formulations with mucin was evaluated through viscosity measurements, as previously described [10]. To determine the interaction with mucin, four solutions were prepared: (1) 5% (*w/w*) mucin suspension, (2) mucin suspension + HA 0.15% (*w/v*) (1:1), (3) mucin suspension + Cr (*w*/*v*) (1:1), and (4) mucin suspension + cHA-Cr-L (*w/v*) (1:1). The muco-adhesion of the formulations was calculated using the following equation:

Δ (%) = [η_muc + sample_ − (η_muc_ + η_sample_)]/(η_muc_ + η_sample_) × 100

where Δ (%) is the muco-adhesion index, η_muc_, η_sample,_ and η_muc + sample_ are the mucin’s, the formulations’, and the mucin with formulations’ viscosity, respectively. For cHA-Cr-L, the mucoadhesive property of η_muc + cHA-Cr-L_ is higher than (η_muc_ + η_cHA-Cr-L_) due to the interactions between the components and mucin [35].

### 4.3. HCEC Exposure to Hyperosmolarity

HCECs were cultured in 12-well plates with explants from corneal limbal rims in a supplemented hormonal epidermal medium (SHEM) containing 5% fetal bovine serum (FBS), according to a previously published method [36]. When they reached confluence, the HCEC cultures were maintained in a serum-free medium and then treated for 4 h with isosmotic (312 mOsM) or hyperosmotic (450 mOsM) medium, which was achieved by adding sodium chloride (NaCl, 69 mM). The medium’s osmolarity was measured by a vapor pressure osmometer (ELITech Group, Torino, Italy). Hyperosmolar HCECs were then treated with cHA-Cr-L, HA, or Cr formulations at 37 °C for 120 min. Gene expression and DCF assays were performed before and after treatments. All experiments were performed in triplicate.

### 4.4. Evaluation of Interleukin-1β (IL-1β) and Tumor Necrosis Factor α (TNFα) Gene Expression by Quantitative Real-Time Polymerase Chain Reaction (RT-qPCR)

Total RNA was extracted from HCECs by RNeasy Plus Mini Kit (Qiagen, Milan, Italy) according to the manufacturer’s instructions and quantified by a spectrophotometer (NanoDrop ND-1000; Thermo Scientific, Wilmington, DE, USA). Reverse transcription from 1 μg of total RNA was performed to synthesize cDNA using Ready-To-Go You-Prime First-Strand Beads as described previously [37]. The real-time PCR was performed (Mx3005PTM system; Stratagene, La Jolla, CA, USA) with a 20 mL reaction volume containing 5 mL of cDNA, 1 mL of TaqMan Gene Expression Assay for IL-1β (Hs01555413_m1) and TNFα (Hs00174128_m1) or GAPDH (Hs99999905_m1) and 10 mL master mix (TaqMan; ABI). The thermocycling was performed at 50 °C for 2 min, 95 °C for 10 min, followed by 40 cycles of 95 °C for 15 s and 60 °C for 1 min. A non-template control was used to evaluate DNA contamination. The results were analyzed by the comparative threshold cycle (CT) method and normalized by GAPDH.

### 4.5. Evaluation of Cellular ROS Production

Cellular ROS production was measured by DCFDA/H2DCFDA—Cellular ROS Assay Kit (Abcam, Milan, Italy) according to the manufacturer’s protocol. DCFDA (2′,7′-dichlorofluorescein diacetate) is a cell-permeable fluorogenic compound that is deacetylated by cellular esterase and subsequently oxidized by ROS to high fluorescent DCF (2′,7′-dichlorofluorescein), which is used to measure cellular ROS levels [37]. HCECs, plated in 12-well plates, were washed with phosphate-buffered saline (PBS) and incubated with 200μL DCFDA (25 μM) solution at 37 °C for 30 min in the dark and rewashed with PBS afterward. Finally, fluorescent intensity was measured by microplate reader using 488 nm for excitation and 535 nm for ROS detection.

### 4.6. Statistical Analysis

Student’s *t*-test was used to compare differences between two groups. One-way ANOVA was used to compare three or more groups, followed by Dunnett’s post hoc test to identify group differences, using SPSS Statistics software version 26.0 (IBM Corp., Armonk, NY, USA). Significant differences were established at *p* < 0.05.

## 5. Summary

The antioxidant and anti-inflammatory effects of cHA-Cr-L on hyperosmolar HCECs suggest that this combined formulation might be a potential approach to treat DED symptoms. Future studies could further investigate the protective effects and mechanism of action of cHA-Cr-L on DED in in vitro and in vivo models. Moreover, clinical studies could evaluate whether this formulation may effectively counteract the deleterious effects of DED in human patients.

## Figures and Tables

**Table 1:** Shear viscosity of cHA-Cr-L compared to linear HA 0.15%.

Formulation	Viscosity [Pa.s] (1[1/s])	Viscosity [Pa.s] (10[1/s])	Viscosity [Pa.s] (100[1/s])
HA 0.15%	0.0033 ± 0.0004	0.0031 ± 0.0001	0.0042 ± 0.0002
cHA-Cr-L	0.0112 ± 0.0006	0.0111 ± 0.0003	0.0099 ± 0.0004

HA, hyaluronic acid; cHA-Cr-L, crosslinked HA-crocin-liposome

## Data Availability

Data is contained within the article.

## Sample Availability

Samples of the compounds are not available from the authors.
